# Perspective: Phase Amplitude Coupling–Based Phase–Dependent Neuromodulation in Parkinson’s Disease

**DOI:** 10.3389/fnins.2020.558967

**Published:** 2020-09-29

**Authors:** Brian Y. Hwang, Yousef Salimpour, Yohannes K. Tsehay, William S. Anderson, Kelly A. Mills

**Affiliations:** ^1^Functional Neurosurgery Laboratory, Division of Functional Neurosurgery, Department of Neurosurgery, Johns Hopkins School of Medicine, Baltimore, MD, United States; ^2^Neuromodulation and Advanced Therapies Clinic, Department of Neurology, Johns Hopkins School of Medicine, Baltimore, MD, United States

**Keywords:** Parkinson’s disease, cortical stimulation, motor cortex, phase-dependent stimulation, phase-amplitude coupling, neuromodulation

## Abstract

Deep brain stimulation (DBS) is an effective surgical therapy for Parkinson’s disease (PD). However, limitations of the DBS systems have led to great interest in adaptive neuromodulation systems that can dynamically adjust stimulation parameters to meet concurrent therapeutic demand. Constant high-frequency motor cortex stimulation has not been remarkably efficacious, which has led to greater focus on modulation of subcortical targets. Understanding of the importance of timing in both cortical and subcortical stimulation has generated an interest in developing more refined, parsimonious stimulation techniques based on critical oscillatory activities of the brain. Concurrently, much effort has been put into identifying biomarkers of both parkinsonian and physiological patterns of neuronal activities to drive next generation of adaptive brain stimulation systems. One such biomarker is beta-gamma phase amplitude coupling (PAC) that is detected in the motor cortex. PAC is strongly correlated with parkinsonian specific motor signs and symptoms and respond to therapies in a dose-dependent manner. PAC may represent the overall state of the parkinsonian motor network and have less instantaneously dynamic fluctuation during movement. These findings raise the possibility of novel neuromodulation paradigms that are potentially less invasiveness than DBS. Successful application of PAC in neuromodulation may necessitate phase-dependent stimulation technique, which aims to deliver precisely timed stimulation pulses to a specific phase to predictably modulate to selectively modulate pathological network activities and behavior in real time. Overcoming current technical challenges can lead to deeper understanding of the parkinsonian pathophysiology and development of novel neuromodulatory therapies with potentially less side-effects and higher therapeutic efficacy.

## Introduction

In Parkinson’s Disease (PD), loss of dopaminergic input into the posterior striatum leads to disordered signaling throughout the basal ganglia-thalamo-cortical (BGTC) network that manifests itself as motor symptoms of bradykinesia, rigidity, and tremor (Hammond et al., 2007). Network-wide synchronized pathological neuronal activity is thought to cause dysfunction at the major nodes of the BGTC network (Bergman et al., 1994; Brown et al., 2001; Bevan, 2002; Hammond et al., 2007). Deep brain stimulation (DBS) is an effective surgical therapy for PD that targets the deep nodes of the BGTC network to ameliorate the pathological neuronal activities. However, current DBS therapies are limited by stimulation induced side-effects and mal-adaptive neuroplasticity from continuous electrical stimulation of the BGTC network at the major nodes (Chen et al., 2006; Tripoliti et al., 2011; Castrioto et al., 2014). Therefore, there has been a significant interest in identifying both parkinsonian and physiologically patterns of neuronal activities that can be used to adjust stimulation parameters in real-time to meet the concurrent therapeutic demand. This closed-loop or adaptive neuromodulation strategy has the potential to significantly improve upon the currently available open-loop DBS systems (Little et al., 2013; Rosa et al., 2015; Little et al., 2016a,b; Piña-Fuentes et al., 2017; Rosa et al., 2017).

Recently, beta-gamma phase-amplitude coupling (PAC) has been identified as a highly promising electrophysiologic biomarker of parkinsonian motor state that can be detected in the superficial node (i.e., the motor cortex) of the BGTC network (de Hemptinne et al., 2013, 2015; Shimamoto et al., 2013; Swann et al., 2015). Unlike the beta activities that represent local information processing, PAC is a type of cross-frequency coupling phenomenon that represents information transmission across multiple cortical and subcortical areas that are involved in both pathologic and normal brain activities such as cognition, perception, and movement (Shimamoto et al., 2013). Excessive PAC is associated with the parkinsonian motor state and can be normalized by both therapeutic dopaminergic medication and DBS therapy in a dose-dependent manner (de Hemptinne et al., 2015; Swann et al., 2015; Wang et al., 2018). These remarkable findings raise the possibility that PAC can serve both as a biomarker to drive adaptive neuromodulation of the deep nodes and as a therapeutic target in an adaptive cortical neuromodulation scheme.

In this article, we review recent evidence regarding PAC in the context of PD and present our perspective on how this biomarker could be leveraged to advance neuromodulation therapies, with an emphasis on utilizing phase-dependent stimulation techniques to target new structures with a lower risk of side-effects.

### Pathological Cortical Electrophysiology in Parkinson’s Disease

Neural oscillations are essential for normal brain processing and are thought to play a critical role in coordinating activities within and across different regions of the brain (Canolty and Knight, 2010; Hyafil et al., 2015; Salimpour and Anderson, 2019). As representations of rhythmic changes in neuronal excitability, oscillations exist in different frequencies across various spatiotemporal scales and have been implicated in attention, cognition, memory, and sensory integration (Gelperin, 2006; Wang, 2010; Watanabe and Hirono, 2016). These synchronous activities are thought to result from simultaneous input from common presynaptic neurons in a tightly regulated manner with each pattern reflecting the timing of separate information processing and integration (Heck et al., 2007). Because of their fundamental importance to the functioning of the brain, unsurprisingly, disturbances in the synchronous patterns have been implicated in various common neurological disorders (Allen et al., 2011; Goutagny et al., 2013; Bahramisharif et al., 2016; Zhang et al., 2017). In PD, a low dopaminergic state classically manifests itself as excessive beta-band power and synchrony throughout the BGTC network (Schnitzler and Gross, 2005; Hammond et al., 2007). However, the electro-pathophysiology of PD is more complicated and may involve multiple functional modes in which there is excessive synchrony both with and between nodes of the BGTC network that are both associated with the severity of certain parkinsonian signs and symptoms (Ahn et al., 2015). This complexity may have resulted from complex interactions that are constantly occurring between neural oscillations across neural circuits and networks.

Indeed, neural oscillations possess unique coupling properties, such as cross-frequency coupling (CFC), in which components of the rhythmic patterns, such as amplitude, phase, and frequency, interact between different frequency bands within and across circuits and networks (Canolty and Knight, 2010). The exact origin of CFC is yet to be elucidated, but transient, mechanistic coupling (i.e., synaptic, electrical) between functionally distinct neuronal subpopulations has been suggested as a potential mechanism (Canolty and Knight, 2010; Salimpour and Anderson, 2019). Phase-amplitude coupling (PAC) is a class of CFC, and it involves coupling between phases of a low-frequency oscillation and the amplitude of a high-frequency oscillation. Low-frequency phases are often dynamically entrained by behaviorally relevant external sensorimotor events and internal cognitive processing implicated in learning, memory, motivation, and decision making (Lakatos et al., 2008; Schroeder and Lakatos, 2009). Therefore, PAC, which represents the modulation of high-frequency power by a low-frequency phase entrained and coordinated with slower external and internal events, is considered a key fundamental mechanism behind cognitive processing (Canolty and Knight, 2010). PAC is generally detected and quantified by first recording raw electrocorticography (ECoG) data from the cortical surface using a subdural electrode, processing the data to estimate the instantaneous power of each frequency band, then calculating the degree of amplitude modulation a by phase measured in modulation index (Tort et al., 2010; Madhavan et al., 2015).

Phase amplitude coupling has been studied in both animals and humans and has been observed across multiple cortical and subcortical sites (Canolty and Knight, 2010; Miller et al., 2012; de Hemptinne et al., 2013, 2015). Implicated in PD is beta-phase and gamma-amplitude coupling in the motor cortex, also known as “beta-gamma PAC.” PAC occurs when the low beta-frequency rhythm synchronizes with the amplitude of gamma oscillations (Miller et al., 2012; Yanagisawa et al., 2012). PAC level in the motor cortex normally fluctuates throughout the movement cycle, such that it elevates in the rest state and decreases in both planning and execution phases of movement (Miller et al., 2012; de Hemptinne et al., 2015). Exaggerated PAC levels are consistently detected in the motor cortex of PD patients both at rest and during movement (de Hemptinne et al., 2013, 2015; Kondylis et al., 2016; Malekmohammadi et al., 2018). Abnormal PAC levels in PD are thought to interfere with the innate CFC processes required for the coordination of action preparation, execution, and alert-rest phases of movement. Importantly, PAC levels increase proportionally to the severity of PD-specific motor symptoms, and therapeutic DBS and medical treatments normalize the PAC levels (de Hemptinne et al., 2015; Swann et al., 2015).

### Cortical Phase-Amplitude Coupling-Based Neuromodulation

**FIGURE 1:**
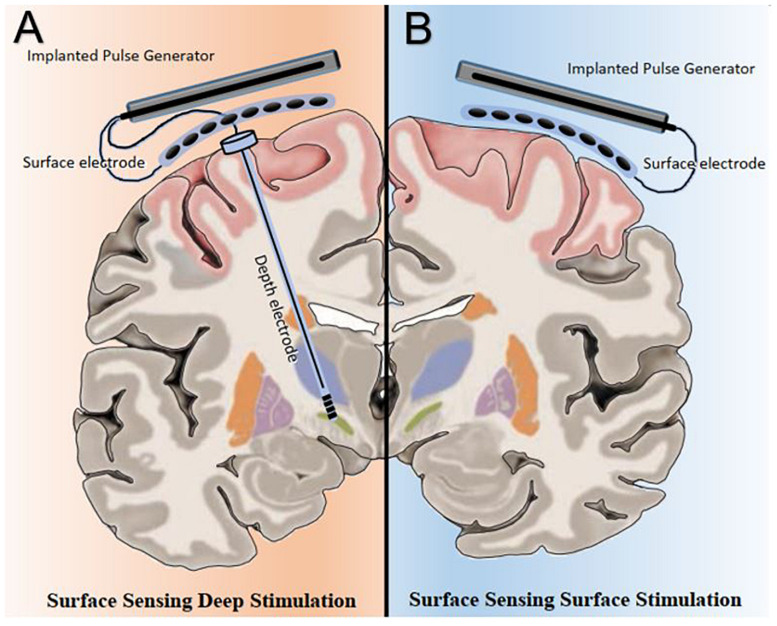
Bata gamma phase amplitude coupling (PAC) levels and patterns measured over the surface of the motor cortex can be utilized in two potential adaptive neuromodulatory strategies. **(A)** In the surface sensing-deep stimulation paradigm, cortical PAC serves as control input to adjust stimulation at the subcortical targets, such as the subthalamic nucleus. **(B)** Under the surface sensing surface stimulation scheme, cortical PAC serves as both feedback and feedforward control signals to drive adaptive motor cortex neuromodulation.

At least two potential novel neuromodulation strategies powered by PAC are possible; surface-sensing deep stimulation, in which PAC drives adaptive neuromodulation of the deep nodes and surface-sensing surface stimulation, in which PAC serves as both the feedback and feedforward control signals in an adaptive cortical neuromodulation scheme (Figure 1). Surface-sensing deep stimulation is a viable strategy that has been attempted with other electrophysiological biomarkers. For example, the cortical gamma-frequency level, which is associated with the severity of dyskinesia, has been successfully used to drive a totally implantable adaptive DBS in PD patients. By means of a subdural cortical lead for sensing, narrow-band gamma power (60–80 Hz) in the motor cortex was used to adjust stimulation amplitudes applied to the subthalamic nucleus (STN) based on patient-specific thresholds (i.e., high and low). The authors demonstrated significantly reduced energy use without worsening clinical symptoms compared to the conventional STN DBS system (Swann et al., 2018). More importantly, they presented a practical and systematic approach to implementing a cortical electrophysiological biomarker to develop a fully implantable adaptive DBS system that future studies could consider. The study also underscored several key advantages of the surface-sensing deep stimulation strategy compared with adaptive DBS systems that sense and stimulate the deep nodes using the same electrodes: (1) good signal-to-noise ratio with minimal stimulation-induced artifact; (2) high fidelity electrophysiological data from subdural electrodes that can yield better quality and quantity of sensory feed forward data than DBS electrodes can; and (3) spatially independent sensing and stimulating elements that minimize inference and optimize placement.

As a cortical biomarker, PAC may also enable the surface-sensing surface-stimulation strategy. The goal of this PAC-based neuromodulation program would be to achieve and maintain a spatiotemporally ‘eu-PAC state’ in the motor cortex and to ameliorate parkinsonian motor symptoms. To develop clinically useful PAC-based neuromodulation strategies, first, a method to detect and modulate PAC level in real time is necessary; and second, pathological and normal (i.e., physiologically and therapeutically) PAC levels and patterns must be defined on a patient-specific basis. The remainder of the article will focus on the PAC-based surface-sensing surface-stimulation strategy, which entails key future challenges in the field of neuromodulation.

### Motor Cortex Stimulation and Phase-Dependent Neuromodulation

As one of the major nodes and an output hub of the BGTC network, the motor cortex may be a high-yield target for neuromodulation. The motor cortex is relatively easy to access, localize, and map compared with the deeper nodes. Its larger size allows precise targeting of symptomatic body regions or movements. Also, concern for uncontrolled current spread to adjacent disparate neuronal populations and fiber tracts is significantly lower compared with the subcortical structures (Maks et al., 2009). Therefore, the motor cortex presents an opportunity for safer and more effective surgical neuromodulation compared to DBS. Nevertheless, motor cortex stimulation has largely been overlooked in neuromodulation research and therapeutics development in PD because of equivocal outcomes associated with cortical stimulation in PD (Canavero et al., 2002; Cilia et al., 2007; Strafella et al., 2007; Moro et al., 2011; De Rose et al., 2012). The unimpressive and variable results of motor cortex stimulation could be related to failing to account for electrophysiologic biomarkers. In all previous cortical stimulation studies in PD, electrical stimulation was delivered to the motor cortex without accounting for the underlying rhythmic activities and state of the BGTC network. A phase-independent approach has a high probability of yielding unpredictable and variable clinical results because the same stimulation pulse applied to the cortex can have a variable effect on PAC levels depending on which part of the beta phase it lands on (Anderson et al., 2009; Cagnan et al., 2013, 2017; Azodi-Avval and Gharabaghi, 2015). Based on network modeling studies, motor cortex neuromodulation should be done adaptively and may need to be based on phase-locked or phase-dependent stimulation techniques to be successful (Anderson et al., 2009; Azodi-Avval and Gharabaghi, 2015).

Phase-dependent stimulation (PDS) is a novel neuromodulation technique with a simple goal: to detect rhythmic brain activity and deliver precisely timed electrical stimulation pulses at a specific phase (Chen et al., 2011; Cagnan et al., 2013; Zrenner et al., 2018; Holt et al., 2019). The potential value of PDS is that, unlike medications or continuous electrical stimulation, it can selectively modulate pathological electrophysiological activities in the disease network without disrupting other physiological processes. The precise and selective nature of PDS may lead to improved symptom control without the stimulation-induced side-effects or maladaptive plasticity associated with continuous stimulation. PDS is relevant to PAC-based surface-sensing surface-stimulation strategy in PD because the technique could deliver the spatiotemporal accuracy and precision required to successfully modulate PAC levels in the motor cortex to meet the therapeutic demand in real time. PDS has been used in healthy animals to selectively and predictably modulate cortical oscillations (Zanos et al., 2018; Kanta et al., 2019; Peles et al., 2020). Zanos et al. demonstrated in non-human primates (NPH) that stimulation triggered from beta oscillations in the sensorimotor cortex not only induces synaptic plasticity, but the direction of the plasticity depends on the stimulated phase of the oscillations. Furthermore, a recent study developed an adaptive neuromodulation system based on a real-time cortical PDS algorithm to show that beta oscillations in the primary motor cortex can be controlled with volition and that PDS can differentially modulate the rhythmic patterns depending on the targeted phase of beta, which in turn, affects behavior (Peles et al., 2020). Similarly, another group used a rat model to adaptively modulate gamma oscillations in the amygdala and successfully affected memory strength (Kanta et al., 2019). The ability of real-time closed-loop PDS to predictably modulate beta oscillations in the subcortical regions in a bidirectional manner has been demonstrated in parkinsonian NPH (Sanabria et al., 2020, Preprint) and rat models (McNamara et al., 2020, Preprint).

Phase-dependent stimulation has also recently been used to selectively suppress pathological synchronized pattern of neuronal firing in the STN of PD patients (Holt et al., 2019). The authors demonstrated that PDS achieved beta suppression without altering the overall firing rate, and the degree of suppression correlated with the number of consecutive single-pulse PDS applied at or near the peak of the beta phase, suggesting that the observed neuromodulatory effect was likely from the PDS-induced alteration in the relative timing of beta activity (Holt et al., 2019). Thus, it is increasingly becoming evident that random or continuous stimulation of cortical and subcortical nodes of the parkinsonian motor network is suboptimal. However, continuous and random stimulation remains the predominant method in research. Future studies should strongly consider the implications of pathological oscillatory patterns in Parkinson’s disease and incorporate PDS as a foundation for neuromodulation.

**FIGURE 2:**
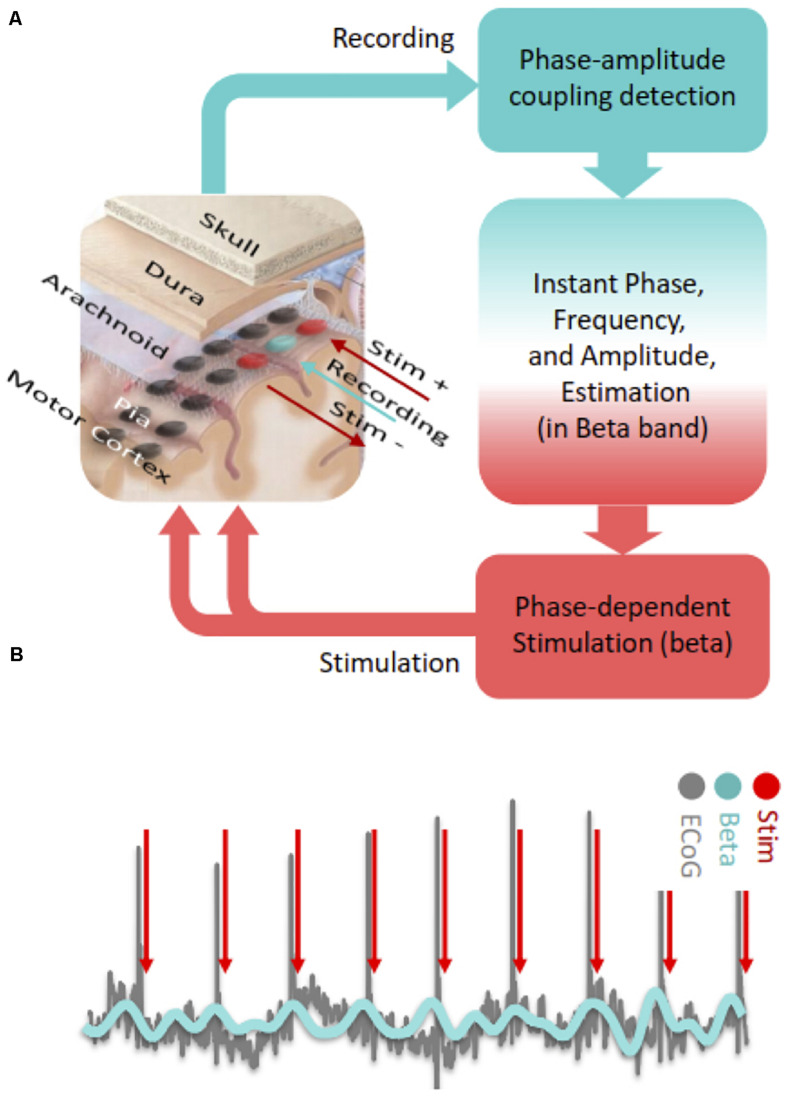
**(A)** Steps of cortical PAC based adaptive phase dependent motor cortex stimulation with implanted subdural grid electrode. In this hypothetical set-up, a single contact (blue) over the motor cortex is used for detecting PAC levels in real-time and two adjacent contacts (red) deliver bipolar phase-dependent stimulation. **(B)** In phase dependent stimulation, electrocorticographic signals recorded from the motor cortex are bandpass filtered in the beta frequency range and electrical stimulation is delivered at a specific phase of the beta oscillation (peak in this case).

With regard to PDS in the motor cortex, it is possible to detect ECoG signals in real time and deliver timed electrical pulses to the cortical region of interest with low latency through fast estimation of phase and frequency of detected oscillations (Chen et al., 2011, 2013; Figure 2A). PAC based neuromodulation techniques can be accomplished through Hilbert transformation-based frequency band optimization, autoregressive (AR) spectral estimation and signal prediction. With this algorithm, it may be possible to detect and modulate PAC in near real-time by targeting specific beta phases (Figure 2B). The AR signal modeling-based approach is robust against signal variabilities and inconsistencies (Chen et al., 2011, 2013). Using this algorithm, we consistently observed acceptable performance in modulating cortical PAC with cortical stimulation in a Parkinson’s patient undergoing awake DBS surgery (Salimpour and Anderson, 2019).

Several critical issues must be considered with regards to a PAC-based PDS protocol. First, evaluation of PAC requires the accumulation of data over several rhythmic cycles, which introduces latency into the stimulation protocol. Latency can be a significant problem in PDS because stimulation-pulse landing off the targeted phase can lead to unintended consequences. Therefore, devising effective strategies to overcome latency problems in PDS protocol is critical. In the context of PD, based on a mean beta-band frequency of approximately 20 Hz, a sliding-time window of 1 s should be sufficient to capture the dynamics of PAC to conduct an effective, near real-time PDS, as demonstrated a recent study (Peles et al., 2020). One solution to mitigating the computational technical demand is to utilize advanced circuit designs based on field-programmable gate array (FPGA), which can provide the speed to handle the high data throughput and processing, flexibility to readily modify algorithms, and machine learning capabilities to continuously improve performance.

Amplitude and time-domain characteristics (e.g., waveform shape) of beta oscillation correlate either with the severity of parkinsonian symptoms or with PAC levels in PD patients and may represent promising alternate biomarkers (Cole et al., 2017; Holt et al., 2019). Because they are technically simpler to work with, beta waveform-shape and amplitude levels may help overcome the latency issue. Nevertheless, we see a value in attempting a PAC-based neuromodulation despite current technical challenges because PAC may better represent the state of the BGTC network in PD. Also, PAC holds several advantages over other electrophysiological biomarkers of PD. First, PAC is strongly associated with parkinsonian symptom severity in a dose-dependent manner and responds predictably to both therapeutic medication and deep brain stimulation. PAC does not require physiological monitoring from deep brain structures, which potentially facilitates a less invasive system overall (de Hemptinne et al., 2013, 2015). Also, human recordings of PAC before, during, and after movement suggest that, compared to other brain disorders, PAC remains elevated even during movement in the parkinsonian state (Blumenfeld et al., 2015; de Hemptinne et al., 2015). We are optimistic that this persists in a stable manner through different movement phases more predictably than local field potential beta power. In comparison, subcortical beta oscillation may be influenced by normal physiological activities more dynamically than PAC and beta power dynamics during movement might now allow for clear thresholding throughout movement initiation and maintenance (de Hemptinne et al., 2015; Holt et al., 2019). Also, the shape of cortical beta oscillations might correlate with motor symptoms and signs of parkinsonism more strongly than the magnitude of beta power from local field potentials, and the atypicality of the waveform shape highly correlates with PAC (Cole et al., 2017). Stability and robustness of various components of the beta waveform shape during movement and against physiological perturbation are yet to be determined. It remains to be seen whether these and other promising biomarkers of PD will outperform PAC in closed-loop neuromodulation. Nevertheless, it is entirely conceivable to develop PAC-based phase-dependent neuromodulation algorithms that concurrently detect and analyze multiple time-domain and spectral features to provide additional controls, such that variables such as waveform shapes and beta amplitudes can either supplement PAC or function as backup biomarkers in failure modes.

Another consideration involves stimulation-related artifact, which can hinder PAC based PDS, particularly in the context of a surface-sensing surface-stimulation paradigm. There are several techniques for reducing stimulation-related artifact both online and offline (Zhou et al., 2018). In our experience, use of separate recording and stimulation electrode contacts can significantly reduce the artifact level (Salimpour and Anderson, 2019). Additionally, the duration of a stimulus artifact is generally very short (3–5 ms), such that online artifact removal techniques can reduce the levels of artifact and noise during stimulation and provide sustained recording.

### Defining Normal Cortical PAC Level as a Therapeutic Goal

For PAC-based neuromodulation to become viable, it will be important to establish PAC patterns that are pathological and physiological for a specific PD patient. Although simple high and low PAC threshold levels can be used to trigger a stimulation pulse, such a system would be too simplistic to perform a dynamically adaptive neuromodulation to achieve the eu-PAC state. Patient-specific normal and pathological patterns would provide the therapeutic windows necessary for a PAC-based PDS neuromodulation system to be clinically safe, effective, and meaningful. Such endeavors have been undertaken with beta activities (Little et al., 2013, 2016a,b; Piña-Fuentes et al., 2017; Rosa et al., 2017). Using an experimental sensing implantable DBS system, beta oscillation patterns associated with specific activities performed outside of the operating room, such as walking and riding a bicycle, have been described (Quinn et al., 2015; Storzer et al., 2017; Syrkin-Nikolau et al., 2017; Hell et al., 2018). Such information is not available for PAC, but the technology exists to safely and chronically record and evaluate PAC patterns from the motor cortex of PD patients (de Hemptinne et al., 2013, 2015). Also, since PAC can be detected non-invasively, characterization of PAC levels and patterns can be achieved during a tuning period in an outpatient setting with minimal risk to guide early artificial intelligence (AI) driven algorithm training prior to electrode array implantation (Swann et al., 2015). Advanced machine learning can be employed to continuously optimize stimulation parameters based on output PAC levels and therapeutic efficacy as defined by the user. AI and machine learning will be critical in the development of next generation of intelligent brain stimulation devices and this topic has been discussed in detail elsewhere (Neumann et al., 2019).

## Discussion

Phase amplitude coupling is expanding our understanding of PD pathophysiology and creating new therapeutic opportunities. Nevertheless, excitement must be tempered as we continue to improve our understanding of the BGTC network and elucidate the relationships among different electrophysiological biomarkers and clinical outcomes. Also, we must continue to define the role of PAC in adaptive neuromodulation and develop creative ways to improve outcomes using the biomarker to define the role of PAC in adaptive neuromodulation and develop creative ways to improve outcomes using the biomarker. Efforts to define physiologically normal and pathological PAC are still needed in order to appropriately gate or modulate stimulation delivery. Going forward, collaboration among various fields, including computational sciences and engineering, is necessary in order to enable data-driven solutions to these remaining hurdles. AI and machine learning will be particularly useful in improving accuracy and speed of PAC detection and pattern recognition. Ultimately, the knowledge, techniques, and technologies that enable PAC neuromodulation may not only improve PD management but also play a significant role in advancing research and therapeutic development in other common neuropsychiatric conditions.

## Data Availability Statement

All datasets generated for this study are included in the article/supplementary material, further inquiries can be directed to the corresponding author.

## Author Contributions

YS and BH equally contributed to the ideas and concepts discussed within this manuscript and played a role in organizing the manuscript and designing the figures discussed within this manuscript. YT contributions include perusing the literature to gather the most recent developments in the field, and assisted in writing some sections of the manuscript. WA and KM provided edits and modifications to the entire manuscript and the references used. All authors contributed to the article and approved the submitted version.

## Conflict of Interest

KM previously received clinical trial support from St. Jude/Abbott and currently receives clinical trial support from Global Kinetics Corporation. WA sits on Advisory Boards for Longeviti Neuro Solutions, and NeuroLogic. WA was also a paid consultant for Globus Medical. The remaining authors declare that the research was conducted in the absence of any commercial or financial relationships that could be construed as a potential conflict of interest.
